# Vitamin B6 deficiency hyperactivates the noradrenergic system, leading to social deficits and cognitive impairment

**DOI:** 10.1038/s41398-021-01381-z

**Published:** 2021-05-03

**Authors:** Kazuya Toriumi, Mitsuhiro Miyashita, Kazuhiro Suzuki, Nao Yamasaki, Misako Yasumura, Yasue Horiuchi, Akane Yoshikawa, Mai Asakura, Noriyoshi Usui, Masanari Itokawa, Makoto Arai

**Affiliations:** 1 Project for Schizophrenia Research, Department of Psychiatry and Behavioral Sciences, Tokyo Metropolitan Institute of Medical Science, Tokyo, 156-8506 Japan; 2 Department of Psychiatry, Tokyo Metropolitan Matsuzawa Hospital, Tokyo, 156-0057 Japan; 3 Department of Psychiatry, Graduate School of Medicine, Shinshu University, Nagano, 390-8621 Japan; 4 Kowa Company Ltd., Tokyo, 189-0022 Japan; 5 Center for Medical Research and Education, Graduate School of Medicine, Osaka University, Osaka, 565-0871 Japan; 6 Department of Neuroscience and Cell Biology, Graduate School of Medicine, Osaka University, Osaka, 565-0871 Japan

**Keywords:** Molecular neuroscience, Schizophrenia

## Abstract

We have reported that a subpopulation of patients with schizophrenia have lower levels of vitamin B_6_ (VB6) in peripheral blood than do healthy controls. In a previous study, we found that VB6 level was inversely proportional to the patient’s positive and negative symptom scale (PANSS) score for measuring symptom severity, suggesting that the loss of VB6 might contribute to the development of schizophrenia symptoms. In the present study, to clarify the relationship between VB6 deficiency and schizophrenia, we generated VB6-deficient (VB6(−)) mice through feeding with a VB6-lacking diet as a mouse model for the subpopulation of schizophrenia patients with VB6 deficiency. After feeding for 4 weeks, plasma VB6 level in VB6(−) mice decreased to 3% of that in control mice. The VB6(−) mice showed social deficits and cognitive impairment. Furthermore, the VB6(−) mice showed a marked increase in 3-methoxy-4-hydroxyphenylglycol (MHPG) in the brain, suggesting enhanced noradrenaline (NA) metabolism in VB6(−) mice. We confirmed the increased NA release in the prefrontal cortex (PFC) and the striatum (STR) of VB6(−) mice through in vivo microdialysis. Moreover, inhibiting the excessive NA release by treatment with VB6 supplementation into the brain and α2A adrenoreceptor agonist guanfacine (GFC) suppressed the increased NA metabolism and ameliorated the behavioral deficits. These findings suggest that the behavioral deficits shown in VB6(−) mice are caused by enhancement of the noradrenergic (NAergic) system.

## Introduction

Vitamin B_6_ (VB6) is a generic name for a family of six interconvertible pyridine compounds: pyridoxal (PL), pyridoxine (PN), pyridoxamine (PM), and their respective 5′-phosphorylated forms (PLP, PNP, and PMP, respectively). PLP is a cofactor for over 150 enzymes (including representatives of every major enzyme class), which account for ~4% of known enzymes^1^. PLP-dependent enzymes are involved in the metabolism of the following neurotransmitters: dopamine (DA), noradrenaline (NA), serotonin (5-HT: 5-hydroxytryptamine), glycine, d-serine, glutamate, γ-aminobutyrate (GABA), and histamine^2^. Thus, it is speculated that VB6 deficiency affects the metabolism of many neurotransmitters, leading to impairments in brain function.

Schizophrenia is a heterogeneous psychiatric disorder characterized by positive symptoms such as hallucinations and delusions, negative symptoms such as apathy and lack of emotion, and cognitive impairment. We have reported that PL levels in peripheral blood of a subpopulation of patients with schizophrenia is significantly lower than that of healthy controls^3–5^. More than 35% of patients with schizophrenia have low levels of PL (clinically defined as male: <6 ng/ml, female: <4 ng/ml). These results have been replicated by other groups^6,7^. PL level is inversely proportional to severity score on the positive and negative symptom scale (PANSS), suggesting that VB6 deficiency might contribute to the development of schizophrenia symptoms^5^. Additionally, we recently reported that high-dose PM was effective in alleviating psychotic symptoms, particularly the PANSS negative and general subscales, in a subset of patients with schizophrenia^8^. Although a link between lower VB6 level and schizophrenia is widely hypothesized, the mechanism behind this remains poorly understood.

In the present study, to investigate the relationship between VB6 deficiency and schizophrenia, we generated VB6-deficient (VB6(−)) mice by feeding with a VB6-lacking diet as a mouse model reflecting the subpopulation of schizophrenia patients with VB6 deficiency. We demonstrated that VB6(−) mice showed hyperactivate noradrenergic (NAergic) signaling, resulting in behavioral deficits comparable to schizophrenia. These are ameliorated by VB6 supplementation into the brain and treatment with the α2A adrenoreceptor agonist guanfacine (GFC), which suppresses NA release.

## Materials and Methods

### Development of VB6-deficient mice

We generated VB6(−) mice by feeding C57BL/6J male mice (Clea Japan Inc, Tokyo, Japan) with a VB6-lacking diet containing 5 μg/100 g VB6 pellets from 8 to 12 weeks of age, while control mice were fed with a normal diet, with 1.4 mg/100 g VB6 pellets. The mice were randomly assigned to the control or the VB6(−) group. They were housed in plastic cages and maintained in a regulated environment (25 ± 1 °C, 50 ± 5% humidity) in a 12 h light/dark cycle (lights switched on at 8 A.M. and off at 8 P.M.). Food and tap water were available ad libitum.

### Measurement of VB6 in mouse plasma and brain tissue

After feeding with the VB6-lacking diet for 4 weeks, mice were sacrificed, and their brains removed. The prefrontal cortex (PFC), hippocampus, nucleus accumbens, and striatum (STR) were dissected on an ice-cold glass plate. Each brain region was rapidly frozen and stored at −80 °C until further analysis.

VB6 concentration in plasma were determined with ID-Vit VB6 (Immunodiagnostik AG: Bensheim, Germany) according to the manufacturer’s protocol. The VB6 score measured by this kit contains all forms of VB6 (PL, PN, and PM) and their phosphorylated forms (PLP, PNP, and PMP).

The concentrations of VB6 in the brain were determined using an Agilent 1260 Infinity HPLC system, which was equipped with an Agilent 1100 series fluorescence detector (Agilent technologies, CA). Briefly, each frozen brain sample was weighed and homogenized with an ultrasonic processor in 28.6 mM acetate buffer (pH 4.5) containing 20% acetonitrile and 2.86% trifluoroacetic acid with 4′-deoxypyridoxine as an internal standard. The homogenates were then placed on ice for 20 min and centrifuged at 9100x*g* for 5 min at 4 °C. The supernatants were filtered using Ultrafree^®^-MC Centrifugal Filter Units (DURAPORE PVDF, 0.22 μm, Merck, Germany) and injected into the HPLC system with a CAPCELL PAK C18 ADME separation column (150 mm × 4.6 mm, Osaka Soda, Osaka, Japan). The mobile phase consisted of a binary gradient elution profile comprising 10 mM ammonium formate buffer (pH 3.0) with 10 mM sodium-1-octanesulfonate and acetonitrile. The flow rate was maintained at a constant of 1 mL/min. The column temperature was set at 40 °C, and the detection wavelength was set at 400 nm with excitation at 290 nm.

### Measurement of monoamine levels in brain

The level of monoamines and their metabolites were determined using a HPLC system, which was equipped with an electrochemical detector (HITEC500; Eicom, Kyoto, Japan) as previously described^9,10^. Each frozen brain sample was weighed and homogenized with an ultrasonic processor in 0.2 M perchloric acid containing isoproterenol as an internal standard. The homogenates were then placed on ice for 30 min and centrifuged at 20,000*xg* for 15 min at 4 °C. The supernatants were mixed with 1 M sodium acetate, pH 3.0, filtrated with 0.22 μm filter (Merck Millipore), and injected into a HPLC system equipped with a reversed-phase ODS column (Eicompak SC-5ODS; 150 mm × 2.1 mm; Eicom) and an electrochemical detector. The column temperature was maintained at 25 °C, and the detector potential was set at +750 mV. The mobile phase was 0.1 M citric acid and 0.1 M sodium acetate, pH 3.6, containing 14% methanol, 180 mg/L sodium-1-octanesulfonate, and 5 mg/L EDTA, and the flow rate was set at 1 mL/min. The monoamine turnover was calculated from the concentrations of each monoamine and their metabolites.

### In vivo microdialysis

After feeding with VB6-lacking diet for 4 weeks, mice were anesthetized with sodium pentobarbital (40 mg/kg, i.p.) and stereotaxically implanted with a dialysis probe (D-I-4-02: 2 mm membrane length; Eicom) in the PFC (AP +1.7 mm, ML −0.3 mm, DV −3.5 mm, from the bregma) and the STR (AP +8.0 mm, ML −1.5 mm, DV −4.0 mm, from the bregma)^11^. The cannula was cemented in place with dental acrylic resin. Microdialysis experiments were conducted the day after surgery. The probe was perfused with Ringer’s solution [147 mM NaCl, 4 mM KCl, and 2.3 mM CaCl_2_ (pH 6.0)] at a constant flow rate of 1 μL/min. A stabilization period of 3 h was established before depolarization stimulation. Microdialysis samples (20 μL) were collected every 20 min and injected immediately onto a HPLC column for simultaneous assaying of monoamines. The concentrations of monoamines in brain microdialysates were determined by HPLC with an electrochemical detector (HTEC-500; Eicom). An Eicompak CAX column (2.0 mm × 200 mm; Eicom) was used, and the potential of the graphite electrode (WE-3G; Eicom) was set to +450 mV against an Ag/AgCl reference electrode. The mobile phase contained 100 mM phosphate buffer (pH 6.0), 500 mg/L sodium-1-octanesulfonate, 50 mg/L EDTA and 30% (vol/vol) methanol, and the flow rate was set at 1 mL/min. Three time points were chosen for measurements to establish baseline levels of extracellular neurotransmitter. For depolarization stimulation, Ringer’s solution containing high K^+^ [51 mM NaCl, 100 mM KCl, and 2.3 mM CaCl_2_ (pH 6.0)] was delivered through the dialysis probe for 20 min to measure the K^+^-evoked release of monoamines.

### Social interaction test

Twelve-week-old male mice were tested for social behavior using the three-chamber social approach paradigm. The apparatus was a square field (W50 × D50 × H40 cm) that is divided into three chambers of equal size by gray walls, which was placed in a dark, sound-attenuated room. During a habituation session, two empty wire cages were placed in the lateral chambers. The mice were placed in the center chamber and allowed to freely explore all chambers for 10 min. Immediately after the habituation session, the mouse was placed in a home cage for 3 min. After the interval, a test session started. The mice were placed in the center chamber again and allowed to freely explore the left and right arenas, which contained a social target (unfamiliar C57BL/6J male mouse) in one chamber and empty in the other chamber. Experimental mice were given 10 min to explore both chambers. The amount of time spent in the lateral chambers and the time spent within 3 cm around the circular wire cage as interaction time was recorded and analyzed by EthoVision tracking system (Noldus, Netherland).

### Novel object recognition test

The novel object recognition test was performed as described previously with minor modifications^12^. The test procedure consisted of three sessions: habituation, training, and retention. Each mouse was individually habituated to the box, with 10 min of exploration in the absence of objects each day for 3 consecutive days (Day 1–3) (habituation session). On day 4, each animal was allowed to explore the box for 10 min, in which two novel objects were placed symmetrically. The time spent exploring each object was recorded (training session). The objects were different in shape and color, but similar in size. Mice were considered to be exploring an object when their heads were facing it or when they were sniffing it at a distance of less than 2 cm and/or touching it with their nose. After the training session, mice were immediately returned to their home cages. On day 5, the animals were placed back into the same box with one of the familiar objects used in the training session and one novel object. Mice were allowed to explore freely for 5 min and the time spent exploring each object was recorded (retention session). The time when the mouse is in contact with the object is checked visually by the observer and recorded as exploratory time. An exploratory preference, the ratio of time spent exploring either of the two objects (training session) or the novel object (retention session) over the total amount of time spent exploring both objects, was used to assess recognition memory.

### Intracerebroventricular treatment with PLP by osmotic pump

After feeding with VB6-lacking diet for 3 weeks, mice were anesthetized with sodium pentobarbital (40 mg/kg, i.p.) and placed in a stereotaxic apparatus. The infusion cannula was connected to a osmotic pump (Alzet 1002; ALZET, CA) filled with PLP in SAL (1.5 ng/μl) and was implanted into the right ventricle (AP +0.45 mm, ML 1.0 mm, DV −2.5 mm, from the bregma and skull)^11^. PLP were continuously infused into the cerebral ventricle at a dose of 9 ng/6 μl per day (flow rate, 0.25 μl/h). Additionally, SAL was used as a control. One week after the start of PLP infusion, novel object recognition test and social interaction test were conducted.

### Guanfacine treatment

GFC (Tokyo Chemical Industry Co., Tokyo, Japan) dissolved in saline (SAL) was administered (1.0 mg/kg/day, s.c.) to VB6(−) and control mice during the last week of feeding with VB6(−) or control diet respectively. Mice were treated with GFC 2 h before the training and retention sessions in the novel object recognition test and the habituation session in the social interaction test.

### Statistical analysis

Statistical differences between two groups were determined with a Student’s *t*-test. Statistical differences between more than three groups were determined using a one-way or two-way ANOVA with repeated measures followed by Bonferroni’s multiple comparison test. A two-sided *F*-test confirmed normally distributed data. The statistical tests were performed using GraphPad Prism version 7.0. Sample size was estimated based on our previous studies. Behavioral experiments were performed by experimenters blind to the group of the animals. Results are reported as mean ± SEM. A value of *p* < 0.05 was considered statistically significant.

### Study approval

In animal studies, the experimental procedures were approved by the Animal Experiment Committee of the Tokyo Metropolitan Institute of Medical Science (approval no. 15001, 16001, 17003, 18006, and 19003).

## Results

### VB6-deficient mice were generated by feeding with VB6-lacking diet

**Fig. 1:**
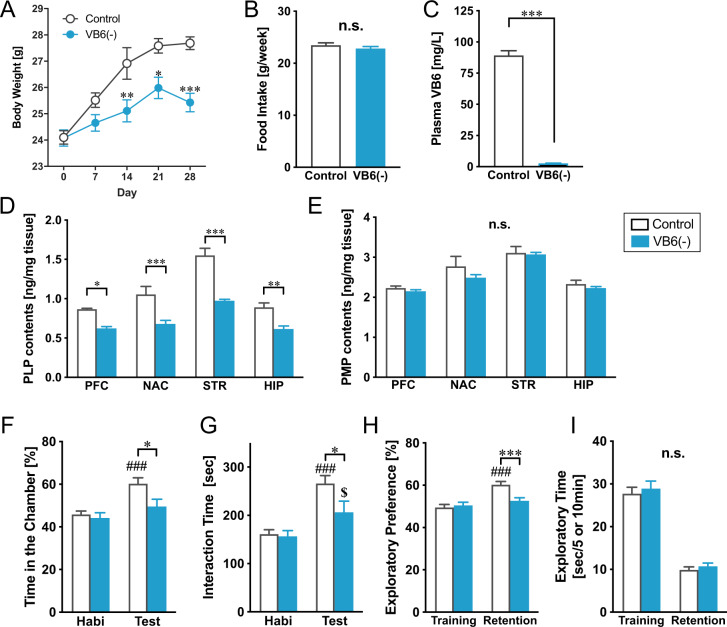
Generation of VB6-deficient mouse model and their behavioral abnormalities. VB6(−) mice were generated by feeding with a VB6-lacking diet from 8 to 12 weeks of age. **A** Changes in body weight during feeding with a VB6-lacking diet are shown. Two-way ANOVA with repeated measurements: *F*_Interaction(4,132)_ = 7.15, *p* *<* 0.001; *F*_Day(4,132)_ = 46.3, *p* < 0.001; *F*_VB6(1,33)_ = 9.82, *p* < 0.01 (Control: *n* = 18; VB6(−): *n* = 17). *p < 0.05, **p < 0.01, and ***p < 0.001 using Bonferroni’s multiple comparison test. **B** Food intake of a control and VB6-lacking diet were measured during the first 1 week of the feeding. n.s. (not significant) using Student’s *t*-test. **C** VB6 level in mouse plasma was determined after feeding for 4 weeks. ***p < 0.001 by Student’s *t*-test. **D** PLP and **E** PMP in the brains of VB6(−) mice were quantified by HPLC. Two-way ANOVA: **D** *F*_Interaction(3,30)_ = 3.58, *p* *<* 0.05; *F*_Area(3,30)_ = 38.0, *p* < 0.001; *F*_VB6(1,10)_ = 76.3, *p* < 0.001 and **E** *F*_Interaction(3,30)_ = 0.53, *p* > 0.05; *F*_Area(3,30)_ = 30.8, *p* < 0.001; *F*_VB6(1,10)_ = 1.32, *p* > 0.05 (*n* = 6). n.s., *p < 0.05, **p < 0.01, and ***p < 0.001 using Bonferroni’s multiple comparison test. In social interaction test, **F** time spent in the chamber and **G** interaction time were measured (Control: *n* = 18; VB6(−): *n* = 17). In the novel object recognition test, **H** exploratory time and **I** exploratory preference were determined (Control: *n* = 20; VB6(−): *n* = 20). Two-way ANOVA: **F** *F*_Interaction(1,33)_ = 4.07, *p* *>* 0.05; *F*_Session(1,33)_ = 19.9, *p* < 0.001; *F*_VB6(1,33)_ = 4.28, *p* < 0.05, **G** *F*_Interaction(1,33)_ = 4.94, *p* *<* 0.05; *F*_Session(1,33)_ = 39.2, *p* < 0.001; *F*_VB6(1,33)_ = 2.88, *p* > 0.05, **H** *F*_Interaction(1,38)_ = 8.10, *p* *<* 0.01; *F*_Session(1,38)_ = 18.2, *p* < 0.001; *F*_VB6(1,38)_ = 5.41, *p* < 0.05, and **I** *F*_Interaction(1,38)_ = 0.02, *p >* 0.05; *F*_Session(1,38)_ = 217, *p* < 0.001; *F*_VB6(1,38)_ = 0.55, *p* > 0.05. *p < 0.05 and ***p < 0.05 using Bonferroni’s multiple comparison test. ^###^*p* < 0.001 and ^$^*p* < 0.05 compared with control and VB6(−) mice in the habituation session respectively, by Bonferroni’s multiple comparison test. The data were represented as mean ± standard error of the mean (SEM). PFC prefrontal cortex, NAC nucleus accumbens, STR striatum, HIP hippocampus.

VB6 is not synthesized de novo in humans, but is primarily obtained from foods^13,14^. We generated VB6(−) mice by feeding with a VB6-lacking diet containing low VB6 at 5 μg/100 g pellets from 8 to 12 weeks of age, while control mice were fed with a normal diet in which VB6 is present at 1.4 mg/100 g. This VB6-lacking diet reduced body growth without a change in food intake (Fig. 1A, B). Moreover, we confirmed that plasma VB6 level in VB6(−) mice [2.62 ± 0.22 ng/ml] decreased to 2.9% of the control group [89.3 ± 3.70 ng/ml] (Fig. 1C). Despite the remarkable reduction in plasma VB6, PLP in the brain of VB6(−) mice was modestly reduced to 60–70% of control level, and PMP in VB6(−) mice was not significantly changed compared with control mice (Fig. 1D, E). Interestingly, PLP and PMP levels were 50–70% and 80–90% of control levels respectively, even after feeding with the VB6-lacking diet for 8 weeks (Supplementary Fig. S1). These findings suggest a mechanism to actively import and maintain VB6 in the brain.

### Behavioral abnormalities in VB6-deficient mice

Next, we assessed behavioral performances of VB6(−) mice to evaluate the effect of VB6 deficiency on schizophrenia-like behaviors. In a social interaction test using a three-chamber apparatus, VB6(−) mice spent less time in the chamber with an unfamiliar mouse and had decreased interaction time during the test session compared with control mice (Fig. 1F, G). Using a novel object recognition test, to investigate recognition memory, VB6(−) mice showed decreased exploratory preference during the retention session compared with control mice, although there was no change in exploratory time (Fig. 1H, I). These data demonstrate that VB6(−) mice display behavioral deficits of sociability and cognitive memory.

### Monoamines in the brain of VB6-deficient mice

**Fig. 2:**
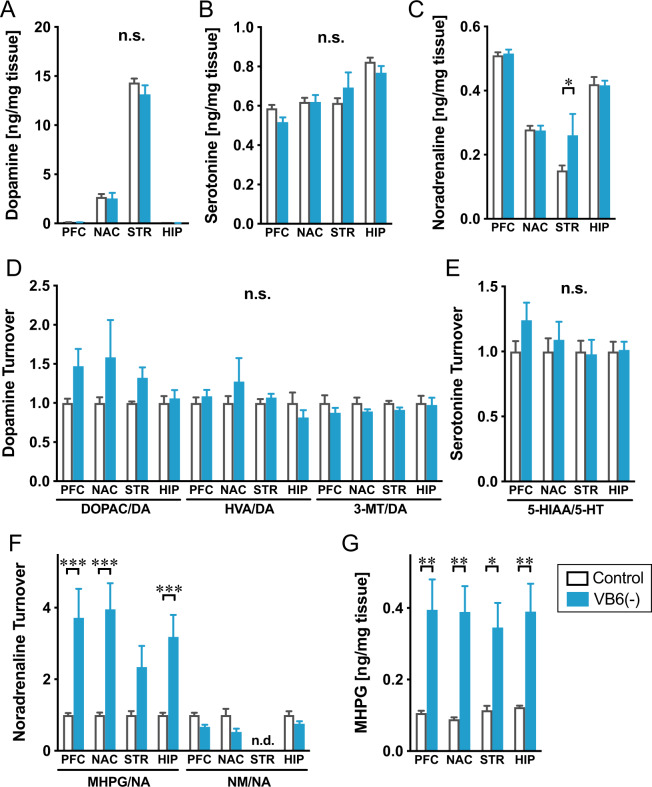
Monoamine levels in the brain of VB6-deficient mice. **A** DA, **B** 5-HT, and **C** NA contents, **D** DA, **E** 5-HT, and **F** NA turnover and **G** MHPG contents in various regions of the mouse brain were determined. *p < 0.05, **p < 0.01 and ***p < 0.001 using Bonferroni’s multiple comparison test (*n* = 6). The data were represented as the mean ± SEM values. n.d. not detected.

To investigate whether VB6 deficiency affects the function of monoaminergic neuronal systems, monoamines and their metabolites were measured in various regions of the brain. Almost no changes were observed in DA, 5-HT, and NA across all brain regions, although NA was increased in the STR of VB6(−) mice (Fig. 2A–C). We also quantified amino acids in the brain of VB6(−) mice and there were almost no changes, except for glutamate, aspartate, and alanine in the STR (Supplementary Fig. 2A–G). Furthermore, to determine functional alterations in the monoaminergic neuronal system, monoamine turnover was evaluated by calculating the ratio of the total tissue monoamine level to monoamine metabolite level. Although there were no changes in DA and 5-HT turnover in all brain regions, NA turnover represented by the ratio of MHPG to NA remarkably increased in the PFC, nucleus accumbens and hippocampus of VB6(−) mice (Fig. 2D–F), which was due to a significant increase of MHPG throughout the brain (Fig. 2G). These findings suggest that the activities of NAergic neuronal systems are enhanced in VB6(−) mice.

### Monoamines release in the PFC and STR of VB6-deficient mice

**Fig. 3:**
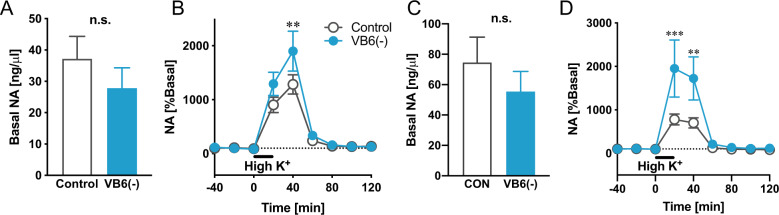
NA release in the PFC and STR of VB6-deficient mice. Basal levels of NA in **A** the PFC and **C** the STR are shown. (n.s. using Student’s *t*-test. *n* = 7). NA release in **B** the PFC and **D** the STR were measured. High K^+^ stimulation was performed at the time point of 0 min (Two-way ANOVA with repeated measures: B *F*_Interaction(8,96)_ = 1.95, *p* > 0.05; *F*_Time(8,96)_ = 46.8, *p* < 0.001; *F*_VB6(1,12)_ = 3.49, *p* > 0.05, **D** *F*_Interaction(8,88)_ = 3.98, *p* < 0.001; *F*_Time(8,88)_ = 19.1, *p* < 0.001; *F*_VB6(1,11)_ = 3.98, *p* > 0.05; **p < 0.01, ***p < 0.001 using Bonferroni’s multiple comparison test. *n* = 7). The data were represented as the mean ± SEM values.

To confirm whether NAergic signaling in VB6(−) mice is altered, we evaluated NA release after high K^+^ exposure using microdialysis. We analyzed the NA release in the STR where the increased NA content was observed and the PFC as a number of reports have suggested that the NAergic neuronal system in the PFC is enhanced in patients with schizophrenia^15–17^. We found no change in basal levels of extracellular NA in both the PFC and the STR of VB6(−) mice (Fig. 3A, C). However, NA release in both the PFC and the STR of VB6(−) mice after high K^+^ stimulation was significantly increased compared to control mice (Fig. 3B, D). These findings suggest that VB6 deficiency activates NAergic signaling widely in the brain, which is consistent with the results indicating an enhanced NA metabolism (Fig. 2F, G).

Furthermore, we also found enhanced DA release in the STR of VB6(−) mice, although no changes in DA release in the PFC and in 5-HT release in both regions (Supplementary Fig. S3). However, we also found that the VB6(−) mice showed no hyperlocomotion and no PPI deficits (Supplementary Fig. S2). Additionally, the VB6(−) mice also showed no change in DA contents and DA turnover in the STR (Fig. 2A, D). These findings suggest that the enhanced DA release in the STR might be insufficient to induce the behavioral deficits in the VB6(−) mice.

### Rescue of behavioral deficits in VB6-deficient mice by PLP supplementation into the brain

**Fig. 4:**
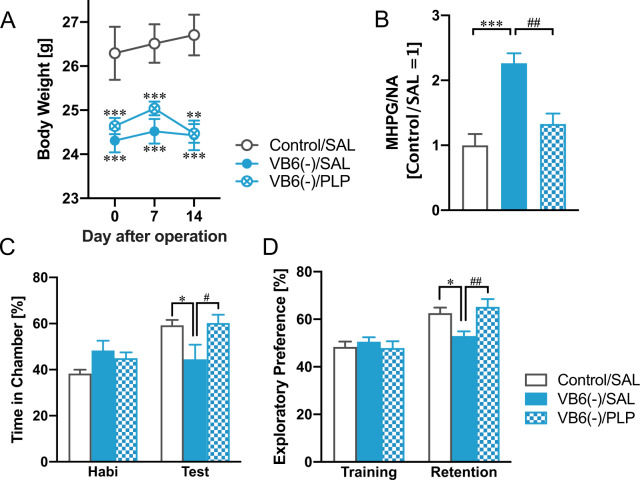
Rescue of behavioral deficits in VB6-deficient mice by PLP supplementation into the brain. **A** Changes in body weight after the implantation of osmotic pump are shown (Two-way ANOVA with repeated measures: *F*_Interaction(4,50)_ = 2.38, *p* > 0.05; *F*_Day(2,50)_ = 3.31, *p* < 0.05; *F*_Group(2,25)_ = 11.6, *p* < 0.05. **B** NA turnover in the PFC were determined by HPLC. One-way ANOVA: *F*_(2,25)_ = 15.7, *p* < 0.001. **C** Exploratory preference in the novel object recognition test and **D** time spent in the chamber in the social interaction test were measured. Two-way ANOVA: **C** *F*_Interaction(2,25)_ = 6.10, *p* < 0.01; *F*_Session(1,25)_ = 38.0, *p* < 0.001; *F*_Group(2,25)_ = 1.67, *p* > 0.05, **D** *F*_Interaction(2,25)_ = 7.67, *p* < 0.01; *F*_Session(1,25)_ = 16.5, *p* < 0.001; *F*_Group(2,25)_ = 1.16, *p* > 0.05. *p < 0.05, **p < 0.01, ***p < 0.001 (vs. control/SAL) and ^#^*p* < 0.05, ^##^*p* < 0.01 (vs. VB6(−)/SAL) using Bonferroni’s multiple comparison test (*n* = 9–10). The data were shown as mean ± SEM values.

Next, to demonstrate whether VB6 supplementation can rescue the NAergic and behavioral changes observed in the VB6-deficient mice and whether the changes are due to VB6 deficiency in the peripheral or central nervous system (CNS), mice were chronically i.c.v. treated with PLP by osmotic pump. The osmotic pump was implanted after feeding with VB6-lacking diet for 3 weeks. One week after the operation, novel object recognition test and social interaction test were conducted. The VB6 supplementation into the CNS suppressed the enhanced NA turnover (Fig. 4B) and improved the behavioral deficits in the VB6(−) mice (Fig. 4C, D), but it could not rescue the reduced body growth (Fig. 4A). These findings suggest that the enhanced NA turnover and the behavioral deficits shown in the VB6-deficient mice are attributed to VB6 deficiency in the CNS, and the reduced body growth is due to that in the peripheral.

### Improvement of behavioral deficits in VB6-deficient mice with GFC treatment

**Fig. 5:**
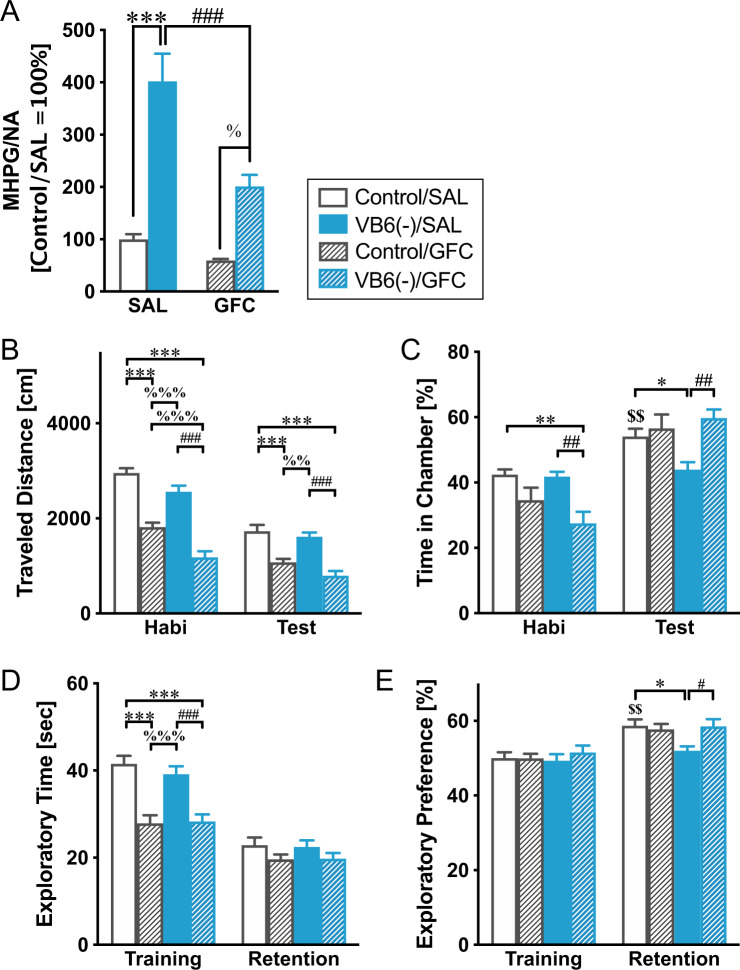
Improvement of behavioral deficits in VB6-deficient mice by guanfacine treatment. VB6(−) mice were administrated GFC during the last of 4 weeks of feeding with VB6-lacking diet. **A** NA turnover in the PFC were determined by HPLC. Two-way ANOVA: *F*_Interaction(1,28)_ = 7.62, *p* < 0.05; *F*_GFC(1,28)_ = 17.3, *p* < 0.001; *F*_VB6(1,28)_ = 58.2, *p* < 0.001. ***p < 0.001, ^###^*p* < 0.001, and ^%^*p* < 0.05 using Bonferroni’s multiple comparison test. In social interaction test, **B** traveled distance and **C** time spent in the chamber were measured (*n* = 12). In the novel object recognition test, **D** exploratory time and **E** exploratory preference were determined (n = 12). Two-way ANOVA: **B** *F*_Interaction(3,44)_ = 6.86, *p* < 0.001; *F*_Session(1,44)_ = 147.8, *p* < 0.001; *F*_Group(3,44)_ = 54.9, *p* < 0.001, **C** *F*_Interaction(3,44)_ = 10.51, *p* < 0.001; *F*_Session(1,44)_ = 71.7, *p* < 0.001; *F*_Group(3,44)_ = 1.30, *p* > 0.05, **D** *F*_Interaction(3,44)_ = 4.78, *p* < 0.01; *F*_Session(1,44)_ = 111.6, *p* < 0.001; *F*_Group(3,44)_ = 17.5, *p* < 0.001, and **E** *F*_Interaction(3,44)_ = 1.07, *p* > 0.05; *F*_Session(1,44)_ = 25.8, *p* < 0.001; *F*_Group(3,44)_ = 4.00, *p* < 0.05. *p < 0.05, ***p < 0.001, ^#^*p* < 0.05, ^##^*p* < 0.01, ^%%^*p* < 0.01, ^%%%^*p* < 0.001, and ^&&&^*p* < 0.001 using Bonferroni’s multiple comparison test. ^$$^*p* < 0.01 compared with control/SAL mice between the habituation and the training session by Bonferroni’s multiple comparison test. The data were shown as mean ± SEM values.

To uncover whether the VB6 deficiency-induced behavioral deficits resulted from enhancement of the NAergic signaling, mice were administrated a selective α2A adrenoreceptor agonist, GFC (which suppresses NA release via stimulation of the α2A autoreceptor in the presynapse), during the last week of the VB6-deficient diet. The sub-chronic GFC treatment normalized NA turnover (Fig. 5A). In the social interaction test, GFC normalized the time spent in the chamber with an unfamiliar mouse in the VB6(−) mice, while GFC also significantly reduced locomotor activity (Fig. 5B, C). Moreover, in the novel object recognition test, GFC normalized exploratory preference in VB6(−) mice. However, the exploratory time in the training session was decreased, probably due to the decrease in locomotor activity (Fig. 5D, E). These results show that the behavioral deficits in VB6(−) mice may be caused by an enhancement of NAergic signaling.

## Discussion

VB6(−) mice were generated through feeding with VB6-lacking diet for 4 weeks and following this, mice showed a remarkable decrease in plasma VB6 level, to ~3% of that in control mice (Fig. 1C). Our previous report found that the mean value of serum VB6 level in healthy subjects is 10.2 ng/ml^5^, which means that 3% of the control levels is ~0.3 ng/ml. However, VB6 level at less than 2.0 ng/ml could not be quantified (not detected) due to a detection limitation in the VB6 measurement system. Several patients with schizophrenia showed “not detected,” indicating that there are at least some patients who have markedly decreased to less than 2.0 ng/ml (<20% control), although our results cannot show the existence of patients whose VB6 has been reduced to 0.3 ng/ml. Additionally, since blood levels of VB6 in control mice are about 8.75 times higher than in humans, it may be difficult to simply compare the deficiency rates between mice and humans.

VB6(−) mice also showed decreased growth without a change in food intake (Fig. 1A, B), which agrees with previous studies^18,19^. This finding suggests that VB6 deficiency induces metabolic abnormalities. In mammals, 70–80% of VB6 in the body is associated with glycogen phosphorylase in the skeletal muscle^20^. VB6 is a cofactor for glycogen phosphorylase in the liver and muscle which catalyzes the sequential phosphorolysis of glycogen to release glucose-1-phosphate^21^. Thus, it is suggested that VB6 deficiency might decrease glucose levels in the peripheral blood via inhibition of glycogen phosphorylase and this may prevent growth. VB6 deficiency was reported to decrease activity of glycogen phosphorylase^22^, and administration of inhibitors against the glycogen phosphorylase reduces glucose level in the blood in humans^23^. The hypothesis is consistent with our findings that the weight loss caused by VB6 deficiency can be explained by its effect on the periphery (Fig. 4A).

Despite the significant reduction in the plasma, PLP, and PMP levels in the brain of VB6(−) mice showed only either a modest decline to 60–70% (PLP) of control levels or no change (PMP) (Fig. 1D, E). Even though feeding with the VB6-lacking diet lasted for 8 weeks, PLP and PMP were decreased to only 50–70% and 80–90% of the control group, respectively (Supplementary Fig. S1). These results imply there is a mechanism to maintain VB6 levels in the brain, although this remains unclear.

VB6(−) mice exhibited impairment in social behavior, cognition and memory comparable with the negative symptoms and cognitive impairment seen in patients with schizophrenia (Fig. 1F–I), which is ameliorated by the α2A receptor agonist GFC (Fig. 5). Furthermore, we found a significant increase in MHPG in whole brain tissue in VB6(−) mice, resulting in a remarkably increased NA turnover (Fig. 2). The enhanced NA release in the PFC and the STR of VB6(−) mice was directly demonstrated by in vivo microdialysis (Fig. 3).

Additionally, we demonstrated that chronic supplementation of VB6 into the CNS by osmotic pump rescued the enhanced NA metabolism and subsequently improved the behavioral deficits in VB6(−) mice (Fig. 4). These findings demonstrate that VB6 deficiency in the CNS, not in the peripheral, increases NAergic signaling in some brain regions, which might induce schizophrenia-like behavioral deficits. As such, the NAergic system may be hyperactivated in patients with schizophrenia who have low VB6 levels, causing negative symptoms and cognitive dysfunction. On the other hand, we found no changes in GABA, DA, and 5-HT in the brains of VB6(−) mice although these neurotransmitters are synthesized by VB6-dependent enzymes (Fig. 2 and Supplementary Fig. S4). These findings may indicate that moderately decreased VB6 in the brain is sufficient to maintain the level of these neurotransmitters. However, modest decline in VB6 is sufficient to impair the NAergic neuronal system, suggesting that the NAergic system might be more vulnerable to VB6 deficiency compared to other neuronal systems. To identify molecular mechanisms underlying our observed finding that VB6 deficiency increases NAergic signaling, further experiments including gene expression analysis would be required on that point.

Early clinical studies have shown that elevated NA signaling plays a pathophysiological role in schizophrenia^15–17^. A number of studies reported that MHPG level in the blood can be used as a biomarker for schizophrenia. MHPG was increased in the blood in patients with schizophrenia compared to healthy subjects, which declined after treatment with neuroleptics^24–26^. A clinical study also showed that NA and MHPG levels correlated both with the severity of negative symptoms and with psychosis ratings in drug-free patients with schizophrenia^27^. In a rodent study, the majority of the plasma MHPG (~83%) is derived from neuronal metabolism of NA^28^, suggesting that the plasma MHPG is largely dependent on neural activity of the NAergic system. Therefore, the increased plasma MHPG observed in patients with schizophrenia implies enhancement of the NAergic system, which is consistent with the findings in the current study. Therefore, similar to MHPG, VB6 level in the peripheral blood might be useful as a biomarker for schizophrenia. In fact, a recent review has shown the decreased VB6 in patients with schizophrenia as the most convincing evidence in peripheral biomarkers for major mental disorders^29^.

High concentrations of NA were reported to cause PFC dysfunction via activation of α1 and β1 adrenoreceptors^30,31^. In fact, microinjection of antagonists for both the α1 and β1 receptor (urapidil and betaxolol, respectively) into the PFC improved cognitive dysfunction induced by hyperactivation of NAergic signaling^30,31^. In the present study, GFC treatment is suggested to inhibit the excessive NA release in the PFC of VB6(−) mice by blockade of presynaptic α2A adrenoreceptors, as demonstrated by normalization of NA turnover (Fig. 5A). Therefore, stimulation of both α1 and β1 receptor might be mitigated by GFC treatment, leading to a normalization of the behavioral deficits seen in untreated VB6(−) mice. In addition, GFC can stimulate the postsynaptic α2A adrenoreceptor as well, which is the pharmacological mechanism of GFC when used therapeutically for attention deficit/hyperactivity disorder (ADHD)^32^. As a result of the effect of GFC on hyperactivity, GFC may have reduced the locomotor activity of VB6(−) mice in the behavioral tests used in this study (Fig. 5B). In contrast to α1 and β1 receptors, many studies have reported that stimulation of the postsynaptic α2A receptor has a beneficial effect on PFC performance^33–42^. Activation of the postsynaptic α2A receptor can prevent the production of cAMP, leading to the closure of hyperpolarization-activated cyclic nucleotide-gated (HCN) cation channels. The blockade of HCN channels in the PFC seems to improve the PFC mediated cognitive performance and strengthens delay-related firing of PFC neurons^43^. As well as suppression of the α1 and β1 receptors resulting from the inhibition of NA release, GFC is suggested to stimulate the postsynaptic α2A receptor, which may also play a role in rescuing the behavioral deficits in VB6(−) mice. It has previously been reported that GFC treatment improved cognitive impairment in patients with schizophrenia^44^ and context processing, a feature of working memory in schizotypal personality disorder^45^. Our findings suggest that GFC might be effective in patients with schizophrenia who have low VB6 levels in peripheral blood. Additionally, supplemental treatment of PM could also be therapeutically beneficial in patients with low VB6, as we reported previously^8^.

The subgroup of the patients with schizophrenia may experience a more prolonged and mild decrease in VB6, and thus the effects on the nervous system may be different. Our VB6(−) mice is a model of acute VB6 depletion, and we believe that it represents the effects of decreased VB6 in a more pronounced manner. In other words, in patients with schizophrenia, the NA system is likely to be more mildly enhanced and the other long-term effects of VB6 deficiency may be arisen additionally. The effects of VB6 deficiency would need to be considered carefully, taking into account the degree and duration of VB6 deficiency.

In conclusion, we have demonstrated by generating VB6(−) mice that VB6 deficiency enhanced NAergic signaling. VB6(−) mice also displayed behavioral impairments, which were ameliorated by VB6 supplementation into the CNS and treatment of α2A adrenoreceptor agonist GFC.

## Supplementary information

Supplementary information
