# Supplementary material for: Vitamin B6 deficiency hyperactivates the noradrenergic system, leading to social deficits and cognitive impairment
Source: Transl Psychiatry. 2021 May 3;11:262. doi: 10.1038/s41398-021-01381-z (PMC8093222; doi:10.1038/s41398-021-01381-z)
Supplement: Supplementary file 1 — Supplementary information [file 41398_2021_1381_MOESM1_ESM.docx]

Supplementary Information for

**Vitamin B6 deficiency activates the noradrenergic system, leading to social deficits and cognitive impairment**

Kazuya Toriumi, Mitsuhiro Miyashita, Kazuhiro Suzuki, Nao Yamasaki, Misako Yasumura, Yasue Horiuchi, Akane Yoshikawa, Mai Asakura, Noriyoshi Usui, Masanari Itokawa, Makoto Arai

Correspondence: [arai-mk@igakuken.or.jp](mailto:arai-mk@igakuken.or.jp) (M.A.)

**This PDF file includes:**

- Supplementary Methods
- Supplementary Figures: S1 to S4

**Supplementary Methods**

***Locomotor activity test***

Locomotor activity was measured using an infrared detector (Muromachi Kikai Co. Ltd., Japan). Mice were placed in a plastic round arena (27cm high, 27 cm in diameter) and locomotor activity was measured over a period of 2 hours.

***Open field test***

The open-field was a square with grey walls (W50 × D50 × H40 cm), placed in a dark, sound-attenuated room. Each mouse was placed in one corner of the open field, and it was allowed to explore the environment freely for 10 min. Time in center (16.7 x 16.7 cm) and traveled distance were determined using the EthoVision system (Neuroscience Idea Co. Ltd., Osaka, Japan).

***Y-maze test***

Short-term memory was assessed by recording spontaneous alternation behavior during a single session in a Y-maze. Each arm was 40 cm long, 12 cm high, 3 cm wide at the bottom, 10 cm wide at the top, and converged in an equilateral triangular central area. Each mouse, naïve to the maze, was placed at the end of one arm and allowed to move freely through the maze during a 10 min session, and the number of arm entries was counted. Each series of arm entries was recorded visually, and an arm entry was defined as when the hind paws of the mouse were completely within the arm. Alternation was defined as successive entries into the three arms on the overlapping triplet sets. The percentage alternation was calculated using the following formula: (number of alternations)/(total number of arm entries-2) × 100 (%).

***Prepulse inhibition (PPI) test***

PPI of the acoustic startle response was measured using an SR-LAB System (San Diego Instruments). The stimulus consisted of a 20-ms prepulse, a 100-ms delay, and then a 40-ms startle pulse. The intensity of the prepulse was 4, 8, or 16-dB above the 70-dB background noise. The amount of PPI was calculated as a percentage of the 120-dB acoustic startle response: 100 – [(startle reactivity on prepulse + startle pulse)/startle reactivity on startle pulse] x 100.

***Forced Swimming Test***

Each mouse was placed in an opaque polyvinyl bucket (24 cm high, 23 cm in diameter), which contained water at 22–23 °C to a depth of 15 cm, and was forced to swim for 10 min. The duration of swimming was measured using an infrared detector (Muromachi Kikai Co. Ltd., Japan). The duration of swimming was measured every minute by an infrared detector. Immobility time was calculated as follows: total time (sec) – swimming time (sec) = immobility time (sec).

**Supplementary Figures**

***Supplementary Figure S1: PLP and PMP in the brain after feeding with VB6-lacking diets for 8weeks***

VB6-deficient mice were generated by feeding with a VB6-lacking diet from 8 to 16 weeks of age. (A) PLP and (B) PMP in the brain of VB6(-) mice were quantified by HPLC. Two-way ANOVA with repeated measurements: (A) *F*_Interaction(3,30)_ = 5.52, *p <* 0.01; *F*_Area(3,30)_ = 4.52, *p* < 0.01; *F*_VB6(1,10)_ = 96.3, *p* < 0.001 and (B) *F*_Interaction(3,30)_ = 1.83, *p* > 0.05; *F*_Area(3,30)_ = 20.0, *p* < 0.001; *F*_VB6(1,10)_ = 64.2, *p* < 0.001 (n = 6). **p* < 0.05, ***p* < 0.01 and ****p* < 0.001 using Bonferroni’s multiple comparison test. The data are represented as mean ± standard error of mean (SEM). PFC: Prefrontal cortex, NAC: Nucleus accumbens, STR: Striatum, HIP: Hippocampus.

******

***Supplementary Figure S2: Behavioral phenotypes of VB6-deficient mice***

In the locomotor activity test, (A) time course and (B) total locomotor activity were determined. Two-way ANOVA: (A) *F*_Interaction(23,759)_ = 0.56, *p >* 0.05; *F*_Time(9,282)_ = 44.4, *p* < 0.0001; *F*_VB6(1,33)_ = 0.35, *p* > 0.05, (B) *F*_Interaction(2,66)_ = 1.20, *p >* 0.05; *F*_Time(2,66)_ = 166, *p* < 0.0001; *F*_VB6(1, 33)_ = 0.35, *p* > 0.05. In the open field test, (C) traveled distance and (D) time spent in the center area were measured. One-way ANOVA: (C) *F*_(1,23)_ =0.73, *p >* 0.05, (D) *F*_(1,23)_ = 0.80, *p >* 0.05. In the Y-maze test, (E) alteration and (F) total arm entry were determined. Student’s t-test: (E) *p =* 0.27, (F) *p =* 0.62. (G) The prepulse inhibition (PPI) score was shown. Two-way ANOVA: *F*_Interaction(2,66)_ = 6.96, *p <* 0.01; *F*_Prepulse(2,66)_ = 133, *p* < 0.0001; *F*_Group(1,33)_ = 0.01, *p* > 0.05. In the forced swimming test, (H) time course of immobilization and (I) total immobility time were determined. Two-way ANOVA: (H) *F*_Interaction(9, 297)_ = 1.02, *p* < 0.01; *F*_Time(5, 167)_ = 70.4, *p* < 0.0001; *F*_VB6(1, 33)_ = 0.02, *p* > 0.05. The data are represented as the mean ± SEM values.

***Supplementary Figure S3: DA and 5-HT release in the PFC and STR of VB6-deficient mice***

Basal levels of DA in (A) the PFC and (E) the STR and 5-HT in (C) the PFC and (G) the STR are shown. (n.s. using Student’s t-test. n = 5-7). DA release in (B) the PFC and (F) the STR and 5-HT release in (D) the PFC and (H) the STR were measured. High K^+^ stimulation was performed at the time point of 0 min (Two-way ANOVA with repeated measures: (B) *F*_Interaction(8,64)_ = 0.54, p > 0.05; *F*_Time(8,64)_ = 18.2, p < 0.001; *F*_VB6(1,8)_ = 0.86, p > 0.05, (D) *F*_Interaction(8,80)_ = 1.84, p > 0.05; *F*_Time(8,80)_ = 19.2, p < 0.001; *F*_VB6(1,10)_ = 0.45, p > 0.05, (F) *F*_Interaction(8,88)_ = 2.15, p < 0.05; *F*_Time(8,88)_ = 20.6, p < 0.001; *F*_VB6(1,11)_ = 2.32, p > 0.05, (H) *F*_Interaction(8,88)_ = 0.31, p > 0.05; *F*_Time(8,88)_ = 19.4, p < 0.001; *F*_VB6(1,11)_ = 0.14, p > 0.05. **p < 0.01 using Bonferroni’s multiple comparison test. n = 5-7). The data are represented as the mean ± SEM values.

***Supplemental Figure S4: Amino acid contents in the brain of VB6-deficient mice***

(A) GABA, (B) glutamic acid, (C) Aspartic acid, (D) Glycine, (E) Glutamine, (F) Taurine and (G) Alanine contents in various regions of the mouse brain were determined by HPLC. **p* < 0.05, ***p* < 0.01, and n.s. using Bonferroni’s multiple comparison test. (n = 12). The data are represented as the mean ± SEM values. n.s.: not significant
